# Association between serum neurofilament light chains and Life’s Essential 8: A cross-sectional analysis

**DOI:** 10.1371/journal.pone.0306315

**Published:** 2025-02-24

**Authors:** Tao Wang, Li-Ming Yan, Teng-Chi Ma, Xiao-Rong Gao

**Affiliations:** 1 Department of Neurology, The Affiliated Hospital of Yan’an University, Yan’an, Shaanxi, China; 2 Department of Gynecology, The Affiliated Hospital of Yan’an University, Yan’an, Shaanxi, China; 3 The First Affiliated Hospital of Xi’an Jiaotong University, Yulin Hospital, Yulin, Shaanxi, China; North Karelia Central Hospital, FINLAND

## Abstract

**Background and aim:**

Serum neurofilament light chain (sNfL), a protein released into the bloodstream post-neuronal axonal damage, has been validated as a robust biomarker for a range of neurological and systemic diseases. Concurrently, Life’s Essential 8 (LE8) comprises a holistic suite of health behaviors and metabolic markers that are essential for assessing and enhancing cardiovascular health. Nevertheless, the interrelation between LE8 and sNfL is not yet fully elucidated. This investigation seeks to evaluate the association between LE8 and sNfL within the framework of the National Health and Nutrition Examination Survey (NHANES).

**Methods:**

According to data from the 2013–2014 NHANES, the study enrolled a total of 5262 participants aged between 20 and 75 years. We excluded 3035 individuals lacking sNfL measurements, included 2071 subjects for analysis, and further excluded cases from LE8 due to missing data. Ultimately, 1691 valid datasets were obtained. Hierarchical and multiple regression analyses were conducted, supplemented by smooth curve fitting and saturation effect analysis to investigate the relationship between LE8 and sNfL.

**Results:**

An inverse correlation was observed between LE8 scores and sNfL levels. For each SD change increase in LE8, log-transformed sNfL levels decreased by 0.14 (-0.17, -0.11 in the non-adjusted model), 0.08 (-0.10, -0.05 in the minimally adjusted model), and 0.08 (-0.12, -0.05 in the fully adjusted model). The multi-factor adjusted β coefficients and 95% confidence intervals (CIs) for LE8 categories (<50, 50 ~ 80, and ≥80) were as follows: reference, -0.20 (-0.34, -0.06), and -0.26 (-0.42, -0.10). The inflection point was determined to be 58.12, identified using a two-piece linear regression model.

**Conclusion:**

The analysis indicated a non-linear relationship between LE8 scores and sNfL levels. Associations were noted a positive association between LE8 and sNfL. These results suggest that lifestyle modifications and optimization of metabolic markers could potentially correlate with reduced sNfL levels; further investigation is necessary to confirm a causal relationship.

## Introduction

Neurofilament, a neuron-specific intermediate filament, comprises light, medium, and heavy chains [1]. Exclusively expressed in mature neurons, neurofilament forms a fibrous network within the cytoskeleton, which provides structural stability and resistance to mechanical stress. Neurofilaments are notably abundant in the axons of large, myelinated neurons, including motor neurons. They play essential roles in axonal and dendritic growth, participate in axonal transport, and serve as scaffolds for microtubules, thereby regulating organelle positioning. Upon neuronal injury or death, neurofilaments are released [2, 3]. Historically, neurofilament research was confined to cerebrospinal fluid (CSF) analyses owing to limitations in detection methods. However, technological advancements have now made it possible to detect neurofilaments in blood samples, which show a high correlation with CSF levels. This breakthrough has significantly enhanced the utility of neurofilament levels as biomarkers for neurological diseases in numerous studies [4, 5]. To date, numerous studies have substantiated the multifaceted roles of CSF and Serum neurofilament light chain (sNfL), elucidating its diagnostic, prognostic, and pharmacodynamic potential across a spectrum of neuroinflammatory, neurodegenerative, cerebrovascular, and traumatic diseases [6–8]. Furthermore, accumulating evidence suggests that sNfL can identify subclinical neuronal damage even in disorders not primarily neurological. Additionally, compelling evidence reveals a complex interplay between cardiovascular risk factors and sNfL levels [9].

In 2010, the American Heart Association (AHA) delineated seven elements—three health behaviors (smoking, diet, physical activity) and four metabolic factors (body mass index, blood pressure, total cholesterol, fasting blood glucose)—aimed at monitoring and promoting cardiovascular health (CVH) across individuals and populations. This comprehensive strategy, termed Life’s Simple 7 (LS7), represented an initiative to enhance heart health [10]. However, with the emergence of additional evidence, the AHA recognized the significance of sleep health as an essential component. In response, a novel metric known as Life’s Essential 8 (LE8) was introduced, quantifying each component on a scale from 0 to 100. LE8 offers a more detailed assessment of health behaviors, metabolic factors, and individual differences, thereby providing advantages in evaluating heart health [11]. Research has also demonstrated that LE8 plays a significant role in enhancing cognitive function and mitigating the risk of dementia. This improvement is likely linked to enhanced cardiovascular function, which, in turn, fosters improved brain function [12–14].

sNfL has been identified as having a complex relationship with a variety of diseases and even all-cause mortality [15]. Nevertheless, the factors that potentially influence sNfL levels and the precise methods to mitigate these levels are still not fully understood. The relationship between LE8 and sNfL has not yet been definitively established, and current research in this area remains insufficient. In our study, we investigated the relationship between LE8 and sNfL, observing that higher LE8 scores correlated with lower sNfL levels. This observation implies that higher scores on the LE8 metric, which includes health behaviors and metabolic markers, could be associated with lower sNfL levels.

## Materials and approaches

### Participants

**Fig 1:**
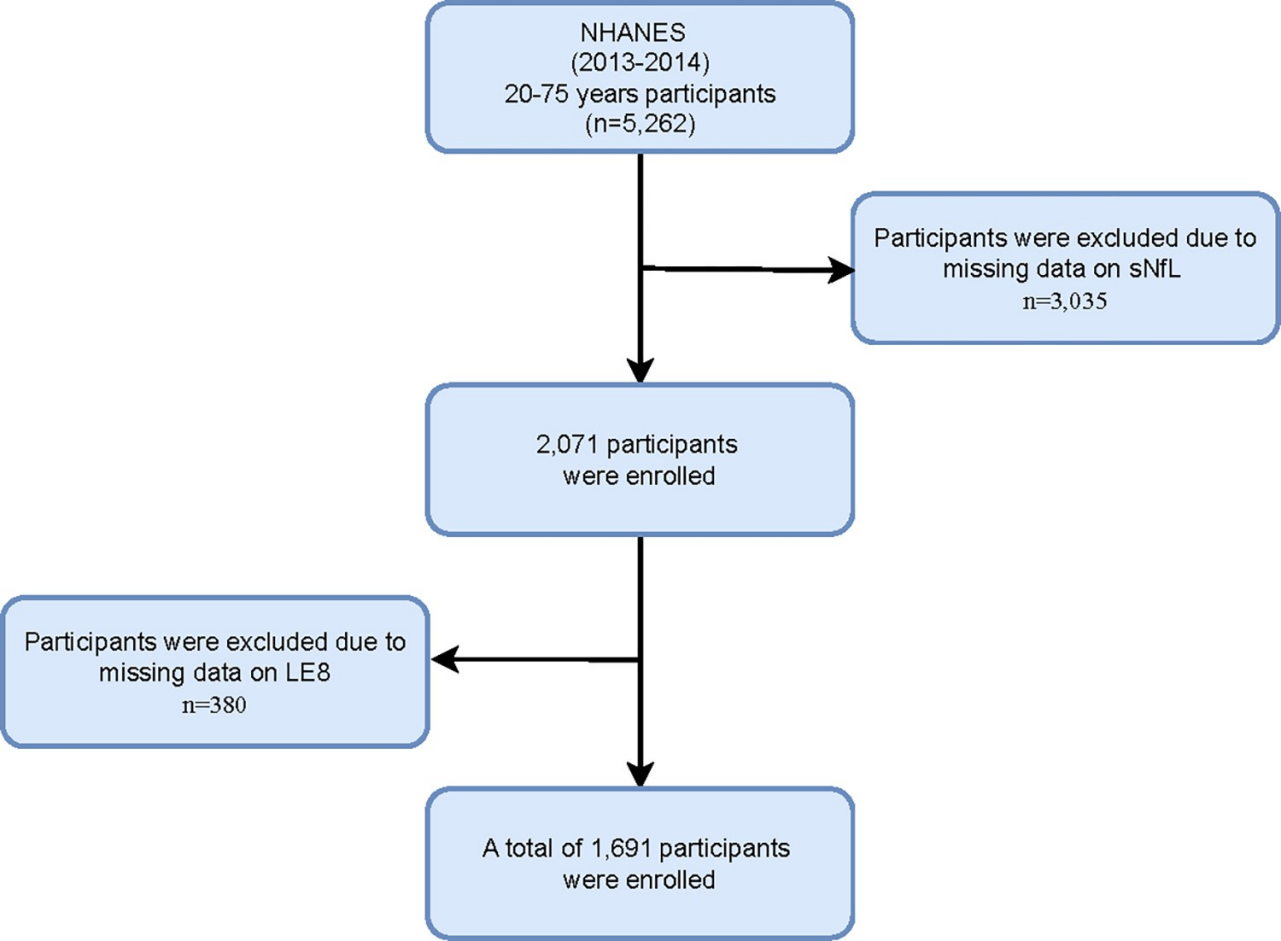
Flow chart of study inclusion legend: Flowchart of the participants’ selection form NHANES 2013–2014.

The National Health and Nutrition Examination Survey (NHANES) is a nationally representative survey conducted by the National Center for Health Statistics (NCHS) of the Centers for Disease Control and Prevention (CDC) in the United States. This survey focuses on the non-institutionalized U.S. resident population and aims to gather comprehensive health and nutrition data. Prior to participation, NHANES ensures that all participants provide informed written consent. NHANES consistent with approval from the National Center for Health Statistics Research Ethics Review Board (NCHS ERB) (Continuation of Protocol #2011–17 for NHANES cycle 2013–2014). In the NHANES 2013–2014 cycle, we included 5,262 participants aged between 20 and 75 years. Of these participants, 5,106 attended a visit at a mobile screening center. For the analysis of sNfL measurements, we excluded 3,035 individuals lacking available data. Ultimately, we collected 2,071 subjects. Then, we calculated the LE8 metric. Consequently, we excluded 380 individuals lacking detailed LE8 information, resulting in a final sample of 1,691 subjects with all valid data. The participant selection process is illustrated in Fig 1.

### Definitions of cardiovascular metrics in LE8

LE8 metric comprises four health behaviors (sleep, nicotine exposure, physical activity (PA), and diet) and four health factors (blood pressure, blood glucose, non-high-density lipoprotein cholesterol (non-HDL), and body mass index (BMI)). Self-reported data included sleep duration, second-hand smoke exposure, combustible tobacco use, minutes per week of physical activity, medication use, diet and history of diabetes. The Healthy Eating Index 2015 (HEI-2015) was utilized to assess the quality of diet. Height, weight, blood pressure, and blood glucose were measured following standardized protocols. BMI was calculated as the ratio of weight (in kilograms) to height (in meters) squared. Systolic and diastolic blood pressures were estimated by averaging the three previous blood pressure measurements. Non-HDL cholesterol was calculated as the difference between total cholesterol and HDL cholesterol. Blood samples were collected and sent to a central laboratory for assessment of fasting blood glucose, lipids, and glycated hemoglobin.

The methods for calculating adult LE8 scores using NHANES data are provided in the Table 1. Each indicator within LE8 ranges from 0 to 100 points, and the overall LE8 score is the average of the eight indicator scores. Higher scores indicate healthier cardiovascular health (CVH). Additionally, the LE8 scores are categorized into three groups: low CVH (scores of 0 to 49), moderate CVH (scores of 50 to 79), and high CVH (scores of 80 to 100) [11, 16].

**Table 1:** Definition and scoring approach for the American Heart Association’s LE8.

Domain	CVH Metric	Measurement	Quantification and Scoring of CVH Metric
Health Behaviors	Diet	Healthy Eating Index-2015 diet score percentile	Quantiles of DASH-style diet adherence **Scoring (Population):** Points Quantile 100 ≥95^th^ percentile (top/ideal diet) 80 75^th^– 94^th^ percentile 50 50^th^– 74^th^ percentile 25 25^th^– 49^th^ percentile 0 1^st^– 24^th^ percentile (bottom/least ideal quartile)
	Physical activity	Self-reported minutes of moderate or vigorous physical activity per week	**Metric:** Minutes of moderate (or greater) intensity activity per week **Scoring:** Points Minutes 100 ≥150 90 120–149 80 90–119 60 60–89 40 30–59 20 1–29 0 0
	Nicotine exposure	Self-reported use of cigarettes or inhaled nicotine- delivery system	**Metric:** Combustible tobacco uses and/or inhaled NDS use; or secondhand smoke exposure **Scoring:** Points Status 100 Never smoker 75 Former smoker, quit ≥5 yrs. 50 Former smoker, quit 1 - <5 yrs. 25 Former smoker, quit <1 year, or currently using inhaled NDS. 0 Current smoker Subtract 20 points (unless score is 0) for living with active indoor smoker in home
	Sleep health.	Self-reported average hours of sleep per night	**Metric:** Average hours of sleep per night **Scoring:** Points Level 100 7 –<9 90 9 –<10 70 6 –<7 40 5 –<6 or ≥10 20 4 –<5 0 <4
Health Factors	Body mass index	Body weight (kg) divided by height squared (m^2^)	**Metric:** Body mass index (kg/m^2^) **Scoring:** Points Level 100 <25 70 25.0–29.9 30 30.0–34.9 15 35.0–39.9 0 ≥40.0
	Blood lipids	Plasma total and HDL-cholesterol with calculation of non-HDL-cholesterol	**Metric:** Non-HDL-cholesterol (mg/dL) **Scoring:** Points Level 100 <130 60 130–159 40 160–189 20 190–219 0 ≥220 If drug-treated level, subtract 20 points
	Blood glucose	Fasting blood glucose or casual hemoglobin A1c	**Metric:** Fasting blood glucose (mg/dL) or Hemoglobin A1c (%) **Scoring:** Points Level 100 No history of diabetes and FBG <100 (or HbA1c < 5.7) 60 No diabetes and FBG 100–125 (or HbA1c 5.7–6.4) (Pre-diabetes) 40 Diabetes with HbA1c <7.0 30 Diabetes with HbA1c 7.0–7.9 20 Diabetes with HbA1c 8.0–8.9 10 Diabetes with Hb A1c 9.0–9.9 0 Diabetes with HbA1c ≥10.0
	Blood pressure	Appropriately measured systolic and diastolic blood pressure	**Metric:** Systolic and diastolic blood pressure (mm Hg) **Scoring:** Points Level 100 <120/<80 (Optimal) 75 120-129/<80 (Elevated) 50 130–139 or 80–89 (Stage I HTN) 25 140–159 or 90–99 0 ≥160 or ≥100 Subtract 20 points if treated level

### Definitions measurement of sNfL

In the 2013–2014 NHANES cycle, sNfL levels were measured using the fully automated Attelica immunoassay system developed by Siemens Healthcare. The detection rate of sNfL was 98.4%. For concentrations below the lower limit of quantitation, an estimate is provided by dividing the lower limit of quantitation by the square root of 2. Analytical methods for sNfL measurement can be accessed on the NHANES website, ensuring transparency, and facilitating reproducibility of the results.

### Covariates

In this study, the calculation of LE8 has considered various variables, including sleep, blood pressure, blood sugar, blood lipids, and BMI. To further analyze the data, we also included age (continuous), gender (male and female), race (Non-Hispanic White, Mexican American, Other Race, Non-Hispanic Black and Other Hispanic), education level (Some college or AA degree, College graduate or above, less than 9th grade, 9-11th grade, High school graduate), and poverty ratio (poor (<1.3), moderate (1.3–3.5), wealthy (>3.5)) as covariates.

### Statistics

All data were combined into a single dataset following the NHANES protocol. Data analysis utilized a stratified sampling approach, and all estimates were calculated using NHANES sample weights, in accordance with NCHS editorial guidelines. Continuous variables are presented as mean ± standard deviation (SD), while categorical variables are presented as frequencies and percentages. First, participants were classified into three groups based on LE8: LE8<50, 50–79, and ≥80. Baseline characteristics were summarized according to the LE8 classification. Differences between groups were compared using the F test for continuous variables and the chi-square test for categorical variables. Secondly, a logarithmic transformation was applied to sNfL. Multiple univariate and multivariate linear regression models were conducted to evaluate the correlations between LE8 and log-transformed sNfL. Multiple linear regression models were used to adjust for covariates, and we present results from unadjusted, minimally adjusted, and fully adjusted analyses. Unadjusted models did not consider any variables. The minimally adjusted analysis (Model Ⅰ) included age, gender, and race as covariates. The fully adjusted analysis (Model Ⅱ) further adjusted for poverty ratio and education level. These covariates were selected based on previous research investigations [17, 18]. To conduct subgroup analysis based on low, medium, and high LE8, hierarchical multiple regression analysis was performed. Then, we analyzed the relationship between each element in LE8 and log-transformed sNfL levels. Finally, to address potential nonlinear relationships, smooth curve fitting was conducted, and the inflection point was calculated using a two-piece linear regression model.

All statistical analyzes were performed using the R statistical software package (http://www.R-project.org, The R Foundation) and EmpowerStats (http://www.empowerstats.com, X&Y Solutions, Inc, Boston, MA). P values less than 0.05 (two-sided) were considered statistically significant.

## Results

### Baseline characteristics of participants

The demographic characteristics of the participants are presented in Table 2. A total of 1691 participants were included, with a mean age of 47.11±15.34. Among them, 789 (46.66%) were males and 902 (53.34%) were females. The majority of participants were non-Hispanic whites, accounting for 761 (45.00%) individuals. The distribution of poverty levels was as follows: 31.22% were classified as poor (<1.3), 31.64% as moderate (1.3–3.5), and 30.93% as wealthy (>3.5). The largest proportion of participants had some college or AA education level, comprising 32.47% of the sample. The participants were classified into three groups based on their LE8 scores: low CVH (0 to 49 points), medium CVH (50 to 79 points), and high CVH (80 to 100 points). Among the participants, 220 (13.01%) were assigned to the low CVH group, 1071 (63.34%) to the medium CVH group, and 400 (23.65%) to the high CVH group. The high-CVH group was characterized by a younger age, a higher proportion of females, a greater representation of non-Hispanic whites, higher wealth levels, and higher education levels. As presented in Table 2, the mean sNfL level was 16.84 ± 21.20. For the sNfL levels, the low CVH group had a mean of 25.42 ± 33.06, the medium CVH group had a mean of 17.38 ± 26.25, and the high CVH group had a mean of 12.59 ± 7.44, respectively.

**Table 2:** Baseline characteristics of the study population.

Characteristics	Total	Low (0–49)	Moderate (50–79)	High (80–100)	P value
N (%)	1691(100)	220 (13.01)	1071 (63.34)	400 (23.65)	
Age (years)	47.11±15.34	50.34±13.17	46.71±15.34	41.23±14.91	<0.0001
sNfL(pg/ml)	16.84±21.20	25.42±33.06	17.38±26.25	12.59±7.44	<0.0001
Sex (%)					0.0036
Male	789(46.66%)	85(42.36%)	540(50.39%)	171(42.72%)	
Female	902(53.34%)	115(57.64%)	531(49.61%)	229(57.28%)	
Race/Ethnicity (%)					<0.0001
Non-Hispanic White	761(45.00%)	140(70.22%)	697(65.07%)	259(64.76%)	
Mexican American	233(13.78%)	11(5.56%)	105(9.82%)	36(9.10%)	
Other Race	230(13.60%)	5(2.64%)	67(6.22%)	49(12.22%)	
Non-Hispanic Black	303(17.92%)	35(17.29%)	143(13.34%)	25(6.25%)	
Other Hispanic	164(9.70%)	9(4.29%)	59(5.54%)	31(7.68%)	
Poverty ratio (%)					<0.0001
<1.3	528(31.22%)	57(28.36%)	285(26.63%)	69(17.15%)	
1.3–3.5	535(31.64%)	90(44.89%)	358(33.46%)	110(27.38%)	
>3.5	523(30.93%)	54(26.76%)	428(39.92%)	222(55.47%)	
Education levels (%)					<0.0001
Some college or AA degree	549(32.47%)	74(36.89%)	374(34.91%)	125)31.26%)	
College graduate or above	461(27.26%)	25(12.68%)	277(25.85%)	210(52.59%)	
Less than 9th grade	109(6.09%)	9(4.52%)	40(3.69%)	12(3.09%)	
9-11th grade	233(13.78%)	40(20.05%)	135(12.63%)	15(3.74%)	
High school graduate	342(20.22%)	52(25.85%)	245(22.91%)	37(9.32%)	
Diet score	39.16±31.42	20.35±22.36	33.35±29.22	55.38±30.13	<0.0001
Physical activity score	71.05±42.02	28.86±42.80	69.55±42.11	95.73±14.55	<0.0001
Nicotine exposure score	70.95±39.35	41.51±42.02	67.35±40.39	93.19±17.07	<0.0001
Sleep health score	81.08±25.09	67.85±30.80	82.10±24.14	90.85±16.98	<0.0001
Body mass index score	58.76±34.55	23.43±23.96	54.10±33.18	83.47±21.42	<0.0001
Blood lipids score	67.39±29.78	45.75±29.22	64.01±29.34	83.87±22.92	<0.0001
Blood glucose score	86.22±25.14	64.59±34.23	89.07±21.93	98.65±7.80	<0.0001
Blood pressure score	71.64±30.67	48.22±31.21	70.17±29.08	91.96±17.28	<0.0001

Data are presented as mean ± SD or n (%)

### Relationships between LE8 and sNfL

We conducted multi-factor regression models to examine the relationship between LE8 and log-transformed sNfL. The results of the regression analysis are presented in Table 3. The crude model presents the unadjusted variables, while Model Ⅰ was adjusted for age, gender, and race. Model Ⅱ was further adjusted for age, gender, race, education level, and poverty ratio. In all models, we observed a negative correlation between LE8 and log-transformed sNfL. When LE8 was treated as a continuous variable, every per SD increase in LE8 was associated with a decrease in log-transformed sNfL by 0.14 (-0.17, -0.11 in the unadjusted model), 0.08 (-0.10, -0.05 in Model Ⅰ), and 0.08 (-0.12, -0.05 in Model Ⅱ), respectively. The multi-factor adjusted β and 95% confidence interval (CI) for log-transformed sNfL levels, compared with the lowest LE8 category, -0.20 (-0.34, -0.06), and -0.26 (-0.42, -0.10), respectively.

**Table 3:** Association between LE8 and log-transformed sNfL (pg/mL).

	Crude Model		Model Ⅰ		Model Ⅱ	
	β (95%CI)	P-value	β (95%CI)	P-value	β (95%CI)	P-value
LE8 per SD change	-0.14 (-0.17, -0.11)	<0.0001	-0.08 (-0.10, -0.05)	<0.0001	-0.08 (-0.12, -0.05)	<0.0001
LE8 category						
Low	Reference		Reference		Reference	
Moderate	-0.25 (-0.40, -0.11)	0.002	-0.19 (-0.32, -0.06)	0.007	-0.20 (-0.34, -0.06)	0.009
High	-0.42 (-0.55, -0.29)	<0.0001	-0.24 (-0.37, -0.12)	0.001	-0.26 (-0.42, -0.10)	0.003

Crude Model: no covariates were adjusted.

Model Ⅰ: age, gender, race/ethnicity were adjusted.

Model Ⅱ: age, gender, race/ethnicity, education level, income to poverty ratio.

Each variable within LE8 was independently analyzed, with the findings systematically outlined in Table 4. Following adjustments for multiple covariates, we discerned significant associations between log-transformed sNfL levels and four specific components of LE8: HEI, PA, smoking status, and glucose levels. We conducted a comprehensive examination of all eight components comprising LE8. By inverting the scoring for indicators of unhealthy lifestyles or biomarkers, we ensured that elevated scores across all components uniformly signified improved lifestyle and health outcomes. Consequently, no conflicting trends were observed in the influence of LE8 on log-transformed sNfL levels.

**Table 4:** Association between LE8 components and log-transformed sNfL (pg/mL).

LE8 components	Crude Model		Model Ⅰ		Model Ⅱ	
	β (95%CI)	P-value	β (95%CI)	P-value	β (95%CI)	P-value
HEI per SD change	0.01 (-0.01,0.03)	0.645	-0.05 (-0.09, -0.01)	0.022	-0.05 (-0.09, -0.01)	0.009
PA per SD change	-0.09 (-0.13, -0.05)	<0.001	-0.04 (-0.08, -0.01)	0.016	-0.04 (-0.08, -0.01)	0.024
Smoke per SD change	-0.04 (-0.09,0.01)	0.073	-0.05 (-0.09, -0.01)	0.005	-0.06 (-0.09, -0.03)	<0.001
Sleep per SD change	-0.03 (-0.09,0.03)	0.315	-0.04 (-0.09,0.01)	0.082	-0.05 (-0.09,0.01)	0.051
BMI per SD change	-0.01 (-0.06, -0.04)	0.707	0.01 (-0.04,0.06)	0.817	0.01 (-0.03,0.05)	0.683
Blood lipids per SD change	-0.06 (-0.11, -0.01)	0.041	0.01 (-0.03,0.03)	0.542	0.01 (-0.04,0.06)	0.560
Glucose per SD change	-0.16 (-0.21, -0.09)	<0.001	-0.08 (-0.12, -0.04)	0.005	-0.08 (-0.13, -0.03)	0.007
Blood pressure per SD change	-0.18 (-0.23, -0.13)	<0.001	-0.06 (-0.11, -0.01)	0.043	-0.06 (-0.13, -0.01)	0.068

Crude Model: no covariates were adjusted.

Model Ⅰ: age, gender, race/ethnicity were adjusted.

Model Ⅱ: age, gender, race/ethnicity, education level, income to poverty ratio.

### The detection of nonlinear relationship

**Fig 2:**
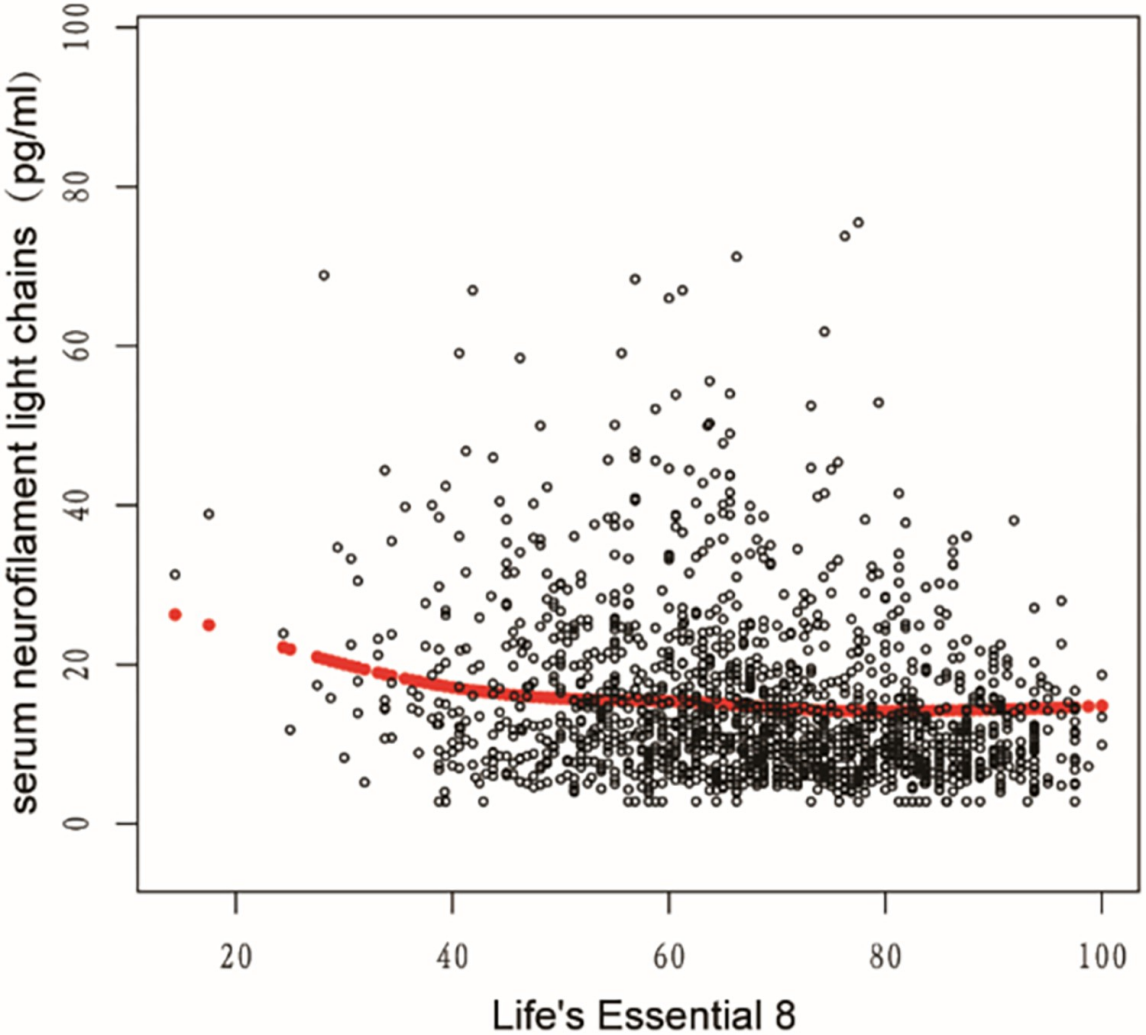
Relationship between LE8 and sNfL. Each black dot represents a sample. Red smooth curve fit indicating the trend. Adjusted for age, gender, race, education, poverty ratio.

**Fig 3:**
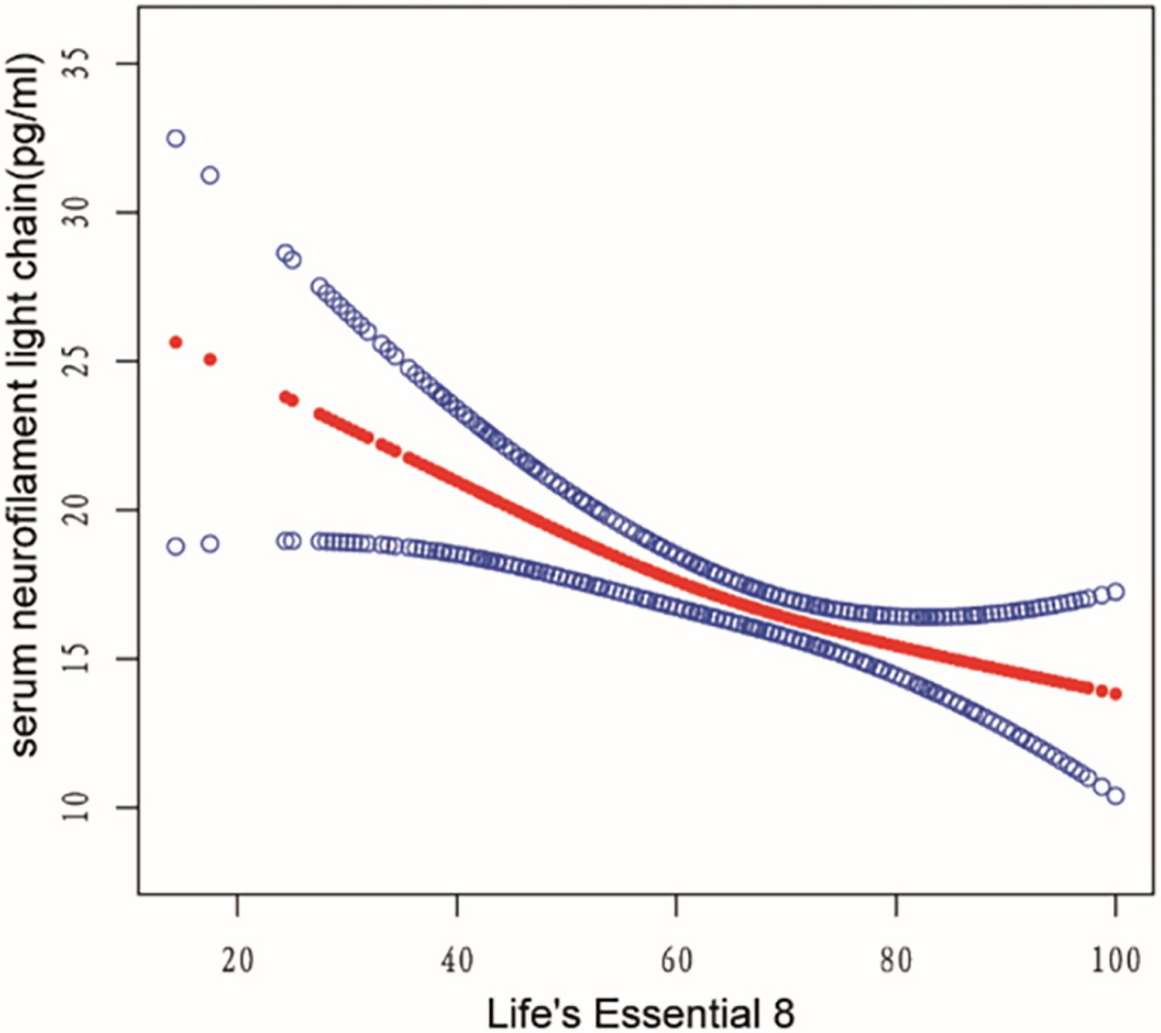
The 95% confidence intervals between LE8 and sNfL. The red points represent the fitting spline; the blue points represent the 95% confidence intervals. Adjusted for age, gender, race, education, poverty ratio.

Utilizing a generalized additive model (GAM) coupled with smooth spline fitting, we identified a pronounced non-linear relationship between LE8 and sNfL, as depicted in Figs 2 and 3. In Fig 2, individual data points are represented as black dots, with a red smooth spline curve illustrating the underlying trend. In Fig 3, the fitting spline is denoted by red points, while the blue points delineate the 95% confidence intervals. The variables adjusted for in this analysis encompass age, gender, race, educational attainment, and poverty ratio.

We calculated the inflection point to be 58.12 using a two-piece linear regression model as presented in Table 5. On the left side of the inflection point, the odds ratio (OR) for LE8 was -0.42 (-0.65, -0.19) with a P value of 0.0004. On the right side of the inflection point, the OR (95% CI) was -0.10 (-0.22, 0.02) with a P value of 0.0935. However, the difference between the two sides of the inflection point was not statistically significant when compared to the linear model (P value for log likelihood ratio test was 0.039).

**Table 5:** The result of two-piecewise linear regression model.

	OR (95%CI)	P value
Fitting model by standard linear regression	-0.19(-0.28, -0.11)	<0.0001
Fitting model by two-piecewise linear regression		
Inflection point of sNfL (pg/ml)	58.12	-
<58.12	-0.42(-0.65, -0.19)	0.0004
≥58.12	-0.10(-0.22,0.02)	0.0935
P for log likelihood ratio test	-	0.039

## Discussion

To the best of our knowledge, this is the first cross-sectional study to investigate the relationship between LE8 and sNfL using a larger sample size. Our study includes a representative sample of the multiracial U.S. population. Firstly, we present demographic data based on LE8 scores (<50, 50–80, ≥80) as stratification. Secondly, we conducted multifactor regression analysis to examine the association between LE8 and Log-transformed sNfL. Our results consistently showed a negative correlation between LE8 and sNfL. Even after adjusting for other variables, we observed that higher LE8 scores were associated with lower sNfL levels. Lastly, our analysis using a nonlinear correlation curve also supports these findings.

Current research is primarily focused on investigating the correlation between sNfL and various diseases. However, there is still no clear consensus on the specific factors that sNfL is associated with. Some individual studies have suggested that sNfL may be linked to age, gender, obesity index, and blood volume, but the exact nature of these relationships has not been fully elucidated [19, 20]. In our study, we aimed to explore the relationship between sNfL and the latest published LE8 factors, which include smoking, diet, physical activity, body mass index, blood pressure, sleep, total cholesterol, and fasting blood glucose. By controlling for a range of confounding factors, we found a negative correlation between sNfL and LE8. Specifically, for every per SD increase in LE8, Log-transformed sNfL levels decreased by 0.14pg/ml. After adjusting for multiple variables, we identified four components—HEI, PA, smoking, and glucose—that showed significant associations with log-transformed sNfL levels.

The extensive application of sNfL has positioned it as a focal point in contemporary neuroscience research. Early studies have demonstrated that smokers generally exhibit elevated levels of sNfL [21]. Additionally, research has indicated that sNfL levels rise with age and are influenced by gender [22]. Building on these findings, future studies should investigate the impact of demographic and physiological variables on blood biomarkers to further understand the broader applicability and significance of sNfL [23]. Several studies have established a multivariate predictive model for sNfL, incorporating variables such as age, creatinine, and glycated hemoglobin (HbA1c) [19]. In the context of Multiple Sclerosis (MS), it has been found that an adapted ketogenic diet (AKD) may reduce sNfL levels independently of relapse activity within the first three months after onset. At six months, the addition of AKD to existing treatments further lowers sNfL levels, suggesting a potential neuroprotective role in the treatment of MS [24]. In studies exploring sNfL, LE8 has demonstrated a more comprehensive association with sNfL.

The application of sNfL is increasingly expanding, with growing evidence supporting its utility in the diagnosis and assessment of drug efficacy in neuroinflammatory conditions such as multiple sclerosis, neurodegenerative diseases like Alzheimer’s disease, cerebrovascular diseases including stroke, and brain trauma [25–29]. Moreover, sNfL may also have the potential to track subclinical neuronal damage and play a unique role in predicting prognosis even in non-primary neurological diseases [30]. Research has shown that sNfL levels significantly increase during and after cardiac surgery, particularly in patients undergoing cardiopulmonary bypass and those experiencing perioperative myocardial injury [31, 32]. Atrial fibrillation and other cardiovascular risk factors have also been found to be closely associated with neurofilament light chain proteins. Patients with atrial fibrillation have higher rates of cognitive impairment and stroke, possibly due to subclinical ischemic stroke or chronic cerebral hypoperfusion [33]. The intricate relationship between cerebral ischemia, hypoperfusion, and neuronal damage may contribute to the pathogenesis of cognitive impairment in populations with higher cardiovascular risk [34]. Increased blood pressure can lead to arteriolar atherosclerosis and small subcortical infarcts, ultimately resulting in elevated levels of neurofilament light chains. Conversely, a rapid decrease in systolic blood pressure may cause cerebral hypoperfusion, a well-known risk factor for accelerated cognitive decline, which could also increase sNfL levels. This proposed mechanism offers a plausible explanation for the observed phenomenon of increased LE8 levels being associated with decreased sNfL levels. Both sNfL and LE8 have demonstrated associations with neurological diseases. Our research has revealed a negative correlation between the two variables. We speculate that combining them to create a new variable may offer greater potential for predicting neurological diseases.

Our study also has certain limitations that should be acknowledged. Firstly, as a cross-sectional study, we cannot establish a causal relationship between sNfL and LE8. The findings would have been more meaningful if we could establish a cause-and-effect relationship. Secondly, the NHANES data collection process also has its limitations. Data on diet, sleep conditions, and physical activities are obtained through recall, which may introduce some discrepancies from the actual situation. Additionally, although we adjusted for potential confounding factors, there may still be residual or unknown confounding effects due to measurement error and unmeasured variables. Lastly, it is important to exercise caution when generalizing the conclusions of this study to other age groups or countries, as our study population consisted of U.S. adults aged 20–75.

## Conclusion

There is a nonlinear relationship between LE8 and sNfL. Associations were noted between lower LE8 scores and decreased sNfL levels. These results suggest that lifestyle modifications and optimization of metabolic markers could potentially correlate with reduced sNfL levels; further investigation is necessary to confirm a causal relationship.

## Supporting information

S1 Table Healthy Eating Index-2015 components & scoring standards. (DOCX)
